# Neurodevelopmental outcomes relevant to autism in juvenile mice exposed to PCB 11 in the maternal diet throughout gestation and lactation

**DOI:** 10.3389/ftox.2026.1816944

**Published:** 2026-08-12

**Authors:** Rebecca J. Wilson, Harmanpreet K. Panesar, Ilknur Dursun, Rosalia Mendieta, Peter M. Andrew, Xueshu Li, Hans-Joachim Lehmler, Pamela J. Lein

**Affiliations:** 1 Department of Molecular Biosciences, University of California Davis, Davis, CA, United States; 2 Department of Physiology, School of Medicine, Istanbul Health and Technology University, Istanbul, Türkiye; 3 Department of Occupational and Environmental Health, University of Iowa, Iowa City, IA, United States

**Keywords:** apoptosis, dendritic arborization, developmental neurotoxicity, glial reactivity, neurodevelopmental disorders, neurogenesis, polychlorinated biphenyls

## Abstract

Polychlorinated biphenyls (PCBs) pose a significant risk to the developing human brain, and recent epidemiological data suggest PCBs increase the risk and/or severity of neurodevelopmental disorders (NDD), including autism spectrum disorder (autism). Experimental animal studies confirm that PCBs disrupt neurodevelopment, resulting in behavioral deficits. Historically, research on the developmental neurotoxicity of PCBs has focused on the higher-chlorinated PCBs found in legacy commercial mixtures; however, lower-chlorinated PCBs (LC-PCBs), including congeners not present in commercial mixtures, predominate in contemporary human exposures. Previous *in vitro* studies demonstrated that one such LC-PCB, PCB 11, altered dendritic arborization, a cellular phenotype common to multiple NDD. Whether PCB 11 modulates dendritic morphogenesis *in vivo* is not known. In this study, we investigated how gestational and lactational exposure to PCB 11 in the maternal diet affected critical neurodevelopmental processes in juvenile male and female mice. C57BL/6J mouse dams were exposed to vehicle or PCB 11 at 0.1 or 1.0 mg/kg/day in their diet for 2 weeks prior to mating and throughout gestation and lactation. Brain tissue was collected from offspring at postnatal day (P)4 and P21 to assess dendritic arborization, apoptosis, glial reactivity, and neurogenesis in the hippocampus and neocortex. PCB 11 dose-dependently reduced the dendritic arborization of pyramidal neurons in the neocortex by 13%–25% but had no significant effect on the dendritic morphogenesis of pyramidal neurons in the hippocampus. Apoptosis was not altered by PCB 11 in either brain region of either sex at either age. While PCB 11 had no effect on neurogenesis at P4, it dose-dependently decreased neurogenesis by 15%–70% in the dentate gyrus at P21. GFAP immunoreactivity and the morphological complexity of astrocytes were increased by 40%–60% and 20%–60%, respectively, in the CA1 hippocampus of female mice exposed to 0.1 mg/kg/day PCB 11; in contrast, PCB 11 had no effect on IBA1 immunoreactivity. These findings demonstrate that developmental exposure to PCB 11 promoted NDD-relevant cellular phenotypes in a dose, sex, and region-dependent manner, providing experimental evidence in support of epidemiological data identifying PCBs as potential NDD risk modifiers. Ongoing studies are investigating behavioral responses in weanling mice developmentally exposed to PCB 11.

## 1 Introduction

Autism spectrum disorder (hereafter referred to as autism) and other neurodevelopmental disorders (NDD) cause lifelong impacts that are costly to the individual, their family, and society (Baxter et al., 2015; Katumba et al., 2023; Shahat and Greco, 2021). This, coupled with evidence that the prevalence of autism has increased over the past several decades (Shaw et al., 2025; Global Burden of Disease Study 2021 Autism Spectrum Collaborators, 2025), has generated considerable interest in identifying factors that influence individual risk and/or severity of autism. Like many NDD, the majority of autism cases cannot be attributed solely to genetic causes, and there is general scientific consensus that most autism arises from complex gene × environment interactions (Hertz-Picciotto et al., 2018; Keil-Stietz and Lein, 2023; Lyall et al., 2017). In contrast to genetic factors, which are currently irreversible, environmental factors are modifiable. Thus, identifying specific environmental factors that influence autism risk may inform strategies for reducing risk of autism and other NDD through public health policies that modify exposure to relevant environmental risk factors.

Narrative and scoping reviews of the peer-reviewed literature that examined links between autism and exposures to environmental pollutants have identified polychlorinated biphenyls (PCBs) as potential environmental risk factors for autism (Carter and Blizard, 2016; Cheroni et al., 2020; Kalkbrenner et al., 2014; Pelch et al., 2019). PCBs are a family of 209 structurally similar compounds, referred to as congeners, that vary according to the number and position of chlorine substitutions on a biphenyl backbone (Ballschmiter et al., 1993). Congeners with four or fewer chlorine substituents are referred to as lower chlorinated PCBs (LC-PCBs); those with more than four chlorine substituents, as higher chlorinated PCBs (HC-PCBs) (Grimm et al., 2015). The toxicological properties of PCBs vary depending on the specific number and location of chlorines and their metabolic transformations (Grimm et al., 2015). PCBs were produced at very high volumes worldwide from the 1930s through the 1980s for use in diverse industrial and commercial applications; however, because of their environmental persistence and growing human health concerns, PCB production was phased out worldwide in the early 2000s (Grimm et al., 2015).

Despite the ban on their production, PCBs continue to be readily detected in contemporary environmental samples, including air in public schools (Grimm et al., 2015; Herrick et al., 2011; Herrick et al., 2016; Marek et al., 2017; Hua et al., 2026), the human food supply (Schecter et al., 2010; Chen et al., 2017; Shin et al., 2015) and human tissues (Megson et al., 2013; Mitchell et al., 2012; Kania-Korwel and Lehmler, 2016). This is due in part to the environmental persistence of the legacy PCBs, which are predominantly HC-PCBs, their continued release from waste sites, PCB-containing fluorescent light ballasts, hydraulic equipment, and capacitors still in use, and the degradation of PCB-containing construction materials used in buildings prior to the ban on PCB production (Klocke and Lein, 2020). In addition, it is now known that PCBs, including congeners that were not present in the legacy Aroclor and other commercial mixtures, are inadvertently produced by contemporary pigment production and other industrial processes (Hu and Hornbuckle, 2010; Anezaki and Nakano, 2014; Herkert et al., 2018). Recent evidence suggests that the inadvertent production of PCBs, many of which are LC congeners, exceeds the peak commercial production of legacy PCBs in the 1970s (Megson et al., 2024).

Epidemiological data support an association between developmental exposure to PCBs and deficits in measures of neuropsychological function in infancy and childhood (Berghuis et al., 2015; Klocke and Lein, 2020; Pessah et al., 2019). More recent human studies have suggested a link between early-life exposures to PCBs and increased risk of NDD (Keil-Stietz and Lein, 2023). While experimental animal studies have confirmed that developmental exposures to PCBs cause behavioral phenotypes and other adverse neurodevelopmental outcomes of relevance to NDD (Breese et al., 2025; Klocke and Lein, 2020), these preclinical studies have focused almost exclusively on the legacy commercial PCB mixtures or HC-PCBs. By comparison, relatively little is known regarding the developmental toxicity of LC-PCBs. This is a concern given evidence that LC-PCBs predominate in contemporary human samples (Koh et al., 2015; Marek et al., 2014). For example, a recent analysis of gestational and neonatal *postmortem* human brains revealed distinct PCB profiles primarily consisting of LC-PCBs at levels reaching as high as 2.8 ng/g wet weight (Li et al., 2022). Furthermore, the LC-PCB congeners 11 and 28 were reported to comprise more than 70% of the total PCBs in the serum of pregnant women at increased risk for having a child with autism (Sethi et al., 2019).

*In vitro* studies found that PCB 11 enhanced dendritic arborization in primary rodent hippocampal and cortical neurons at concentrations as low as 1fM (Sethi et al., 2017a). The shape, number and branching pattern of the dendritic arbor are critical determinants of neuronal connectivity and network function, and altered dendritic arborization is a convergent cellular phenotype observed across multiple NDD, including autism (Lamanna and Meldolesi, 2024; Copf, 2016). Disruption of normal dendritic morphogenesis suggests a plausible biological mechanism to support the proposed association between PCBs and increased NDD risk; however, it has yet to be determined whether PCB 11 interferes with normal dendritic morphogenesis in the intact developing brain. Here, we tested the hypothesis that gestational and lactational exposure to PCB 11 in the maternal diet affects dendritic morphogenesis, apoptosis, neurogenesis, and/or neuroimmune cell reactivity. Modulation of the timing, spatial distribution and/or magnitude of these neurodevelopmental processes have been implicated in the etiopathogenesis of many NDD, including autism (Ghiani and Faundez, 2017). Notably, these neurodevelopmental processes are highly sensitive to environmental insults during gestation and early postnatal life and often exhibit sex- and brain region-specific vulnerability (Keil-Stietz and Lein, 2023; Lein et al., 2026; McCarthy, 2020). This study provides causal evidence that PCB 11 interferes with neurodevelopmental processes known to be altered in NDD, including autism.

## 2 Materials and methods

### 2.1 Materials

Organic peanut butter (Trader Joe’s, Monrovia, CA, United States) and organic peanut oil (Spectrum Organic Products, LLC, Melville, NY, United States) were purchased from Trader Joe’s (Davis, CA, United States). 3,3ʹ-Dichlorobiphenyl (PCB 11; CAS: 2050-67-1; >99% purity) was synthesized by diazotization of 3,3-dichlorobyphenyl with sodium nitrite, followed by reduction with hypophosphorous acid. PCB 11 was characterized by ^1^H-NMR, ^13^C-NMR, and GC-MS in the Synthesis Core of the Iowa Superfund Research Program (The University of Iowa, Iowa, United States) (Holland et al., 2017). All PCB stock solutions were made in dry sterile dimethyl sulfoxide (DMSO, Sigma-Aldrich, St. Louis, MO, United States) (Sethi et al., 2017a). PCB 11 was dissolved in peanut oil and stored in amber glass vials at −20 °C as a stock concentration of 20 mg/mL. A 12.5 µL or 125 µL aliquot of 20 mg/mL peanut oil/PCB stock was mixed into 10 g of peanut butter using a bullet blender (Model: BB50-DX, Next Advance, Inc., Averill Park, NY, United States) for 3 min.

### 2.2 Animals

All procedures involving animals were conducted in accordance with the NIH Guide for the Care and Use of Laboratory Animals and were approved by the University of California, Davis Institutional Animal Care and Use Committee. C57BL/6J mice were purchased from Jackson Labs (Sacramento, CA, United States). All animals were housed in clear polysulfone plastic shoebox cages containing corn cob bedding and maintained on a 12 h light and dark cycle at 22 °C ± 2 °C with 40%–50% humidity. Feed (Diet 5058, LabDiet, Saint Louis, MO, United States) and water from polysulfone plastic water bottles with metal sippers were available *ad libitum*.

### 2.3 Gestational and lactational exposure

Adult female C57BL/6J mice (8–10 weeks old) were randomly divided into one of three experimental groups using a random number generator (GraphPad Prism 8 software- San Diego, CA, RRID:SCR_002798): (1) vehicle (peanut oil in peanut butter); (2) PCB 11 at 0.1 mg/kg/day; or (3) PCB 11 at 1.0 mg/kg/day. Doses were chosen to match those used in earlier studies of mice that exhibited dendritic arborization (Keil Stietz et al., 2021) and behavioral deficits (Sethi et al., 2021) following developmental exposure to the human-relevant MARBLES PCB mix that contains 24% PCB 11 (Sethi et al., 2019). Our goal was to achieve PCB levels in mice that compare to LC-PCB blood levels observed in women for the MARBLES cohort (Sethi et al., 2019; Sethi et al., 2017a) and approximate LC-PCB levels observed in human neonatal brain (Li et al., 2022). All animals were weighed daily prior to dosing and the amount of PCB 11 in peanut butter was adjusted accordingly to account for weight changes throughout pregnancy and lactation.

Two weeks prior to mating, previously unmated dams (>8 weeks of age) were dual-housed, and PCB dosing of dams was initiated. PCB dosing of the dams continued until pups reached postnatal day (P) 21. Pups were never directly dosed. Each dam was individually placed in a clean cage with the PCB-peanut butter mixture in a clean weigh boat and monitored to ensure the mixture was fully consumed before they were placed back in the home cage. During mating, an age-matched un-exposed male was housed with two females for 7 days. During this period, females were checked daily for the presence of a copulatory plug, which was considered gestational day 0. After mating, dams were singly housed throughout gestation and with their pups after parturition. At P2, pups were culled or cross-fostered within dose-matched litters born the same day to ensure all litters consisted of 6–8 pups. One male and one female pup from each litter were euthanized on P4 to collect brains for analyses of caspase-3 activity and TdT-mediated dUTP nick end labelling (TUNEL) staining. Dams and remaining pups were euthanized on P21 to collect brains for Golgi staining, TUNEL staining, and immunohistochemical analyses. Euthanasia was conducted by anesthetizing animals with 5% isoflurane in medical grade oxygen (Airgas Healthcare LC, Radnor, PA, United States) and medical grade compressed air (Airgas Healthcare LC, Radnor, PA, United States) followed by euthanasia by exsanguination via transcardial perfusion with ice-cold 1X phosphate-buffered saline (PBS; 137 mM NaCl, 10 mM sodium phosphate dibasic, 1.8 mM potassium phosphate monobasic pH 7.2) at a rate of 10.5 mL/min using a Masterflex peristaltic pump (Cole Palmer, Vernon Hills, IL, United States).

### 2.4 Brain tissue preparation

Brains were harvested rapidly and bilaterally dissected on ice. One brain hemisphere was microdissected to isolate the cortex and hippocampus, which were then flash frozen on dry ice and stored at −80 °C for caspase-3 analysis. The remaining brain hemisphere was blocked into 2-mm thick sections using a stainless-steel small mouse brain matrix (Kent Scientific, Torrington, CT, United States) and post-fixed in cold 4% w/v paraformaldehyde (Sigma) in 1X PBS for 24 h at 4 °C. Tissue sections were then incubated overnight in 30% w/v sucrose (Thermo Fisher, Waltham, MA, United States) in 1X PBS at 4 °C and subsequently embedded in OCT embedding medium (Thermo Fisher) and stored at −80 °C. All brain sections were cryosectioned at −20 °C into 10 µm slices on Superfrost Plus slides (Thermo Fisher).

For all endpoints examined in intact brain sections, a minimum of two slides per region per animal were analyzed, as previously described (Guignet et al., 2020); however, in most cases, three to four sections spanning approximately 600 µm were analyzed. In cases where sections were excluded due to tissue damage or staining artifacts, remaining sections that met quality criteria were used for analysis. Sections were spaced at least 50 µm apart to avoid oversampling adjacent tissue.

TUNEL staining and all immunohistochemical biomarkers, except those used to assess neurogenesis, were examined in the somatosensory cortex and the CA1, CA3, and dentate gyrus subregions of the hippocampus; neurogenesis biomarkers were assessed in the subgranular zone (SGZ) and granular cell layer (GCL) of dentate gyrus. Anatomical regions were defined with reference to a standard mouse brain atlas (Paxinos and Franklin, 2013), and sections corresponding to defined stereotaxic coordinates (relative to bregma; ∼ −1.34 mm to −1.82 mm) were selected. For each experiment, equivalent anatomical levels were analyzed across all animals to ensure consistency between experimental groups.

### 2.5 Golgi staining

Golgi staining was conducted as previously described (Keil Stietz et al., 2021). Briefly, brains from P21 mice were carefully dissected from the skull immediately following euthanasia, and the brains were processed using the FD Rapid GolgiStain kit (catalog # PK401; FD NeuroTechnologies, Columbia, MD, United States) according to the manufacturer’s instructions. Brains were sectioned into 100 µm thick coronal sections using a Leica VT 1000S vibratome (Microsystems Inc., IL, United States), placed onto gelatin-subbed glass slides (Fisher, Catalog # 12-550-15), and further processed using reagents provided in the FD Rapid GolgiStain kit according to the manufacturer’s protocol. The Golgi-stained brain sections were imaged by confocal microscopy (Olympus IX-81 inverted confocal microscope; Olympus, Shinjuku, Japan) at a magnification of 40X using the MetaMorph Advanced image analysis software (version 7.1, Molecular Devices, Sunnyvale, CA, United States) to acquire the images. Stacks (100–200 images per neuron spaced 0.2 µm apart) of brightfield images of pyramidal neurons were captured from the hippocampus (CA1 region) and the neocortex (layers V/VI of the cortex). Neurolucida software (version 11, MBF Bioscience, Williston, VT, United States) was used to trace basilar dendritic arbors. All tracings were performed by a single individual who was blinded to the identity of the experimental group. Individual neurons were selected for dendritic analysis based on previously described criteria (Wilson et al., 2017). Briefly, these criteria included: (a) neurons whose dendritic arbors did not overlap the dendritic arbors of adjacent neurons; (b) neurons with clearly distinguishable dendritic tips, (c) the dendritic arbor exhibited a pyramidal shape. Neurolucida Explorer software (version 11, MBF Bioscience) was used to quantify dendritic arbor complexity and to perform Sholl analysis in which a series of concentric Sholl rings spaced 10-µm apart were centered on the neuronal soma and the number of dendrites that crossed each ring was counted. At least five neurons per animal from at least eight females and eight males per group from eight different litters (e.g., one female and one male per litter) were assessed.

### 2.6 Caspase-3 assay

Caspase-3 activity in brain homogenates was measured using a fluorometric assay kit (Abcam, catalog # ab39383, Cambridge, United Kingdom). Tissues were thawed and homogenized using plastic pestles with a Kimble Pellet Pestle (DWK Life Sciences, Rockwood, TN, United States) in lysis buffer supplied with the kit (1:3 ratio of weight of tissue (g): amount of lysis buffer (mL)) and incubated on ice for 10 min. After centrifugation for 10 min at 10,000 × *g*, supernatants were transferred to clean tubes and assayed for protein levels using a Pierce BCA kit (ThermoSci, catalog #23227). Remaining supernatant was diluted to a concentration of 2 μg/μL and assayed for caspase activity based on fluorometric detection (Ex/Em 400/505 nm) of free 7-amino-4-trifluoromethyl coumarin (AFC). Fold-increase in caspase 3 activity was determined by comparing samples from PCB-exposed animals to sex and age-matched controls.

### 2.7 TUNEL staining

TUNEL staining was performed using the DeadEnd Fluorometric TUNEL System (Promega, catalog # G3250; Madison, WI, United States) according to the manufacturer’s instructions using solutions provided in the kit. Briefly, following a 5-min wash in 1X PBS, slides were immersed in 4% PFA for 15 min. Slides were washed twice with 1X PBS, and a 20 μg/mL Proteinase K solution (provided with the kit) was added to each tissue section and incubated for 9 min. The slides were washed for 5 min in 1X PBS and then immersed in 4% PFA for 5 min. Following an additional wash in 1X PBS, the slides were laid horizontally in a humidification chamber and incubated in 100 μL of equilibration buffer (provided in the kit) at room temperature for 7 min. During this time, a positive control slide was isolated, equilibrated in DNase I buffer (provided in the kit) for 5 min, and then incubated with 100 μL of DNase I working solution (20 μg/mL) for 10 min at room temperature. The positive control was then washed four times with deionized water for 2 min each and then incubated with equilibration buffer in the humidification chamber following the same protocol used for the remaining slides. Following the equilibration buffer, slides were incubated in 50 µL of nucleotide reaction mixture (provided in the kit), covered with a plastic coverslip to evenly distribute the solution, and laid horizontally in the humidified slide chamber for 1 h. The negative control slide was incubated in a nucleotide reaction mixture that did not contain rTdT enzyme (provided in the kit) to prevent reactivity. The slides were then immersed in a 2X SSC stop solution (provided in kit) for 15 min, washed three times in 1X PBS, and then mounted in Prolong Gold Antifade Mounting solution with DAPI (Invitrogen, catalog # P36931). Once dry, slides were visualized at ×20 magnification using the FITC channel on a high-content ImageXpress XL imaging system (Molecular Devices, Sunnyvale, CA, United States). Regions of interest (ROI) were created for the somatosensory cortex and hippocampus, including the CA1, CA3, and dentate gyrus subregions, as confirmed using a mouse brain atlas (Lein et al., 2007).

### 2.8 Immunohistochemistry

Immunohistochemistry experiments were performed as previously described (Supasai et al., 2020; Andrew et al., 2024). Briefly, antigen retrieval was performed using 10 mM sodium citrate buffer (pH 6.0) in distilled water at 90 °C for 30 min. To block nonspecific binding, slides were incubated in a blocking buffer comprised of 10% v/v normal goat serum (Vector Laboratories, Burlingame, CA, United States), 1% w/v bovine serum albumin (Sigma), and 0.03% w/v Triton X-100 (Thermo Fisher) in 1X PBS for 1 h at room temperature. Following blocking, slides were incubated overnight at 4 °C with primary antibodies in blocking buffer. Primary antibodies used included markers for neurogenesis, specifically neuronal nuclei (NeuN) (Millipore; ABN78; 1:750; RRID:AB_10807945), Ki67 (Abcam, AB15580; 1:500; RRID:AB_443209), and Doublecortin (DCX) (Millipore; AB2253; 1:500; RRID:AB_1586992); astrocytes, including glial fibrillary acidic protein (GFAP) (Synaptic Systems; 173-308; 1:1,000; RRID:AB_2905596) and s100β (Abcam; AB52642; 1:1,000; RRID:AB_882426); and microglia, specifically, ionized calcium binding adaptor molecule 1 (Iba1) (Wako; 019-19741; 1:500; RRID:AB-839504) and CD68 (AbD Serotec Biorad; MCA 1957; 1:200; RRID:AB-322219). Negative controls incubated in blocking buffer in the absence of a primary antibody were included in each staining batch.

Following primary antibody incubation, slides were washed three times in 1X PBS with 0.03% w/v Triton X-100 for 5 min and subsequently incubated in secondary antibody solution for 90 min at room temperature in the dark. Secondary antibodies utilized for the neurogenesis markers include Goat anti-rabbit IgG (Invitrogen; A11034; 1:500; AF488; RRID:AB_2576217) and Goat anti-guinea pig IgG (Invitrogen; A21450; 1:1000; AF647; RRID:AB_2535867). Secondary antibodies for astrocytic markers include Goat anti-guinea pig IgG (Invitrogen; A11073; 1:1000; AF488; RRID:AB_2534117) and Goat anti-Rabbit IgG (Invitrogen; A11036; 1:1000; AF568; RRID:AB_10563566). Secondary antibodies for microglial markers include Goat anti-rabbit IgG (Invitrogen; A11036; 1:1000; AF568; RRID:AB_10563566) and Goat anti-rat IgG (Invitrogen; A11006; 1:1000; AF488; RRID:AB_2534074). Following incubation, slides were rinsed three times in 1X PBS with 0.03% w/v Triton X-100 for 5 min each and mounted in 50 µL ProLong Gold Antifade Mountant with DAPI (Invitrogen).

Slides were scanned at ×20 magnification using a high-content ImageXpress XL imaging system (Molecular Devices, Sunnyvale, CA, United States). ROI were created manually for the somatosensory cortex and hippocampus, including the CA1, CA3, and dentate gyrus subregions, between approximately −1.34 mm to −1.82 mm posterior to bregma. Bregma ranges were confirmed using a mouse brain atlas (Lein et al., 2007). Fluorescent images were quantified as previously described (Supasai et al., 2020). For quantification of astrocytes, microglia, and neurogenesis, cell counts were expressed as cell density (cells/mm^2^) by normalizing counts to the measured area of the manually defined region of interest. This normalization accounts for minor differences in ROI size resulting from image cropping.

### 2.9 Morphometric analyses of astrocytes

Multiple morphological parameters readily quantifiable from 2D images were assessed in GFAP-immunopositive cells (Delgado-García et al., 2024; Baldwin et al., 2024). Colocalization with DAPI was used to identify individual astrocytes. Morphological analysis was performed using custom macros developed in ImageJ (Fiji distribution). Images were first split into DAPI and GFAP channels, followed by background subtraction and automated thresholding to generate binary masks. GFAP+ cells were segmented using particle analysis, with size and circularity filters applied to exclude artifacts. For each segmented GFAP+ cell, standard shape descriptors (area, perimeter, circularity, solidity) were computed directly from the binary mask. To quantify branching morphology, GFAP+ cells were skeletonized and analyzed using skeleton-based algorithms to extract the number of branches, junctions, and branch lengths. Convex hull area was estimated from the relationship between measured area and solidity. All steps were automated via custom macros available on GitHub (version-controlled) and archived on Zenodo (DOI: 10.5281/zenodo.19716216), enabling reproducible batch processing of 2D fluorescence images.

### 2.10 Statistics

Litter was treated as a random effect for all analyses. Statistical analysis was performed using JMP Pro (Cary, NC, United States, RRID: SCR_014242) and GraphPad Prism software (version 8, San Diego, CA, United States, RRID:SCR_002798). The dendritic morphology of Golgi-stained neurons was analyzed as previously described (Wilson et al., 2017; Keil et al., 2017). The mixed model procedure in JMP Pro was used to perform a mixed effects analysis to account for the hierarchical structure of the data. Neurons were nested within animals and animals were nested within litters to account for clustering at both levels. Random intercepts were included for both animals and litters. Sex and dose were treated as fixed effects. Covariance structure for all models was specified under the variance component of this model, and for Sholl analysis, autoregressive [AR (1)] covariance structure was used due to multi-level nesting. For Sholl profiles, the area under the curve (AUC), distance from soma of the peak number of dendritic intersections (Peak X), and maximum number of dendritic intersections (Peak Y) were calculated using the GraphPad Prism Software (n = 8 litters, one male and one female per litter, data reported as mean ± SEM). For caspase assay analysis, the data were assessed for normality using Shapiro-Wilk Test and comparisons were done with a one-way analysis of variance (ANOVA) followed by Tukey’s multiple comparison test for parametric data or Kruskal–Wallis test with Dunn’s multiple comparison test for nonparametric data. These analyses were performed using GraphPad Prism software (version 10.4.1). Outcomes for TUNEL staining were quantified as region-specific positive-labelled cells with 75% overlap with DAPI staining using a custom Fiji macro. Two-way ANOVA followed by Tukey’s multiple comparison test was used to compare between dose groups and sexes using GraphPad Prism software (version 10.4.1). Immunostaining was quantified as region-specific positive-labelling or region-specific mean fluorescence intensity (DCX staining only) using custom Fiji macros. Data were analyzed using a two-way ANOVA followed by *post hoc* Tukey’s multiple comparison test using GraphPad Prism software (version 10.4.1). For all data, *P-*values <0.05 were considered significant. All analysis was completed by an individual blinded to the experimental groups.

## 3 Results

### 3.1 PCB 11 did not cause overt maternal or fetal toxicity

**FIGURE 1:**
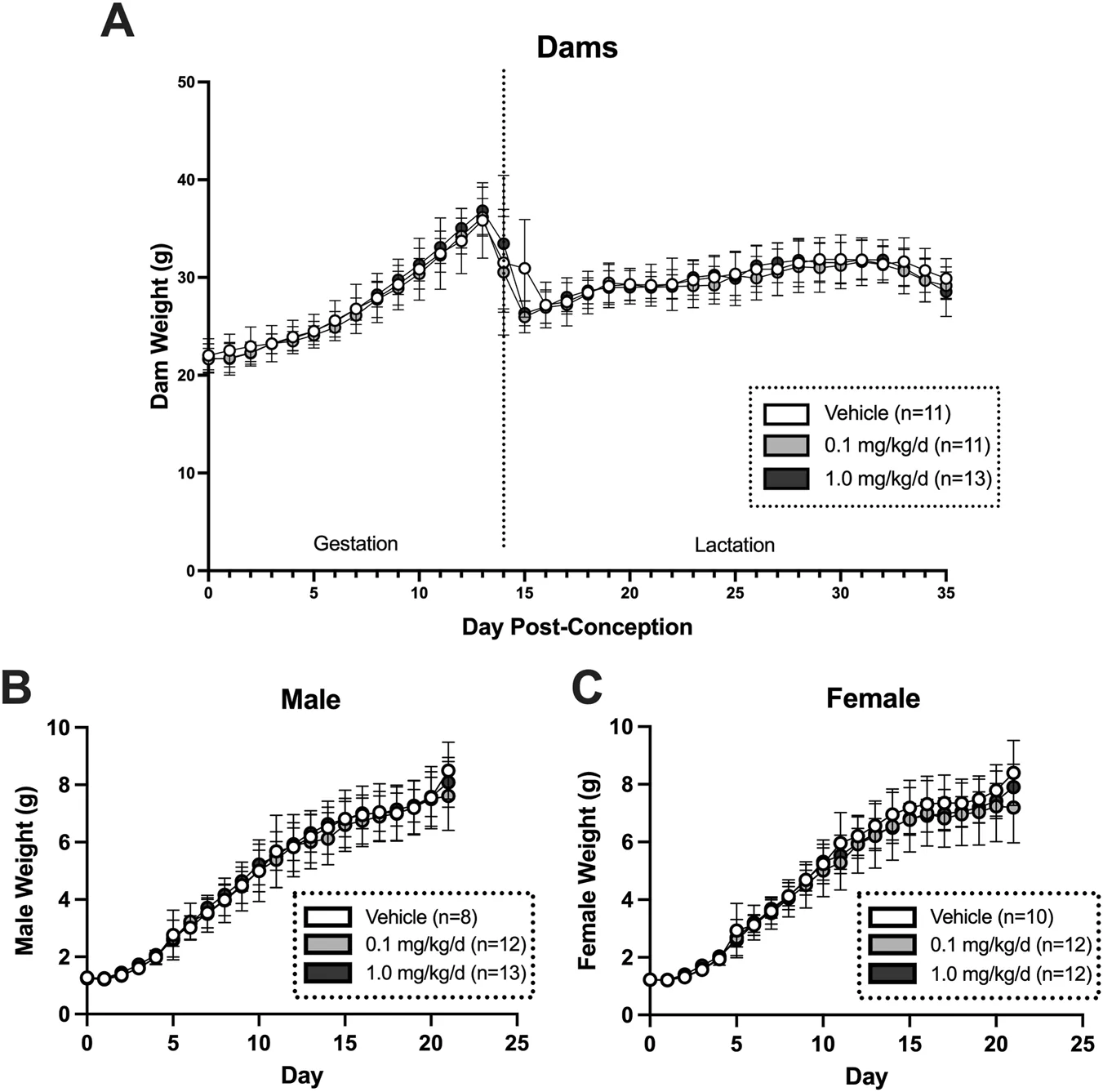
PCB 11 had no significant effect on dam or pup weight throughout the exposure period. **(A)** Dam weights were measured daily starting at conception and continuing throughout gestation and lactation until pups were weaned (n = 11–13 animals per group). Weight of **(B)** male and **(C)** female pups was recorded daily from birth until P21 when pups were weaned (n = 8–13 animals per group). Data are presented as the group mean (dot) ± SD (bars). Data were analyzed using mixed effects modeling.

**FIGURE 2:**
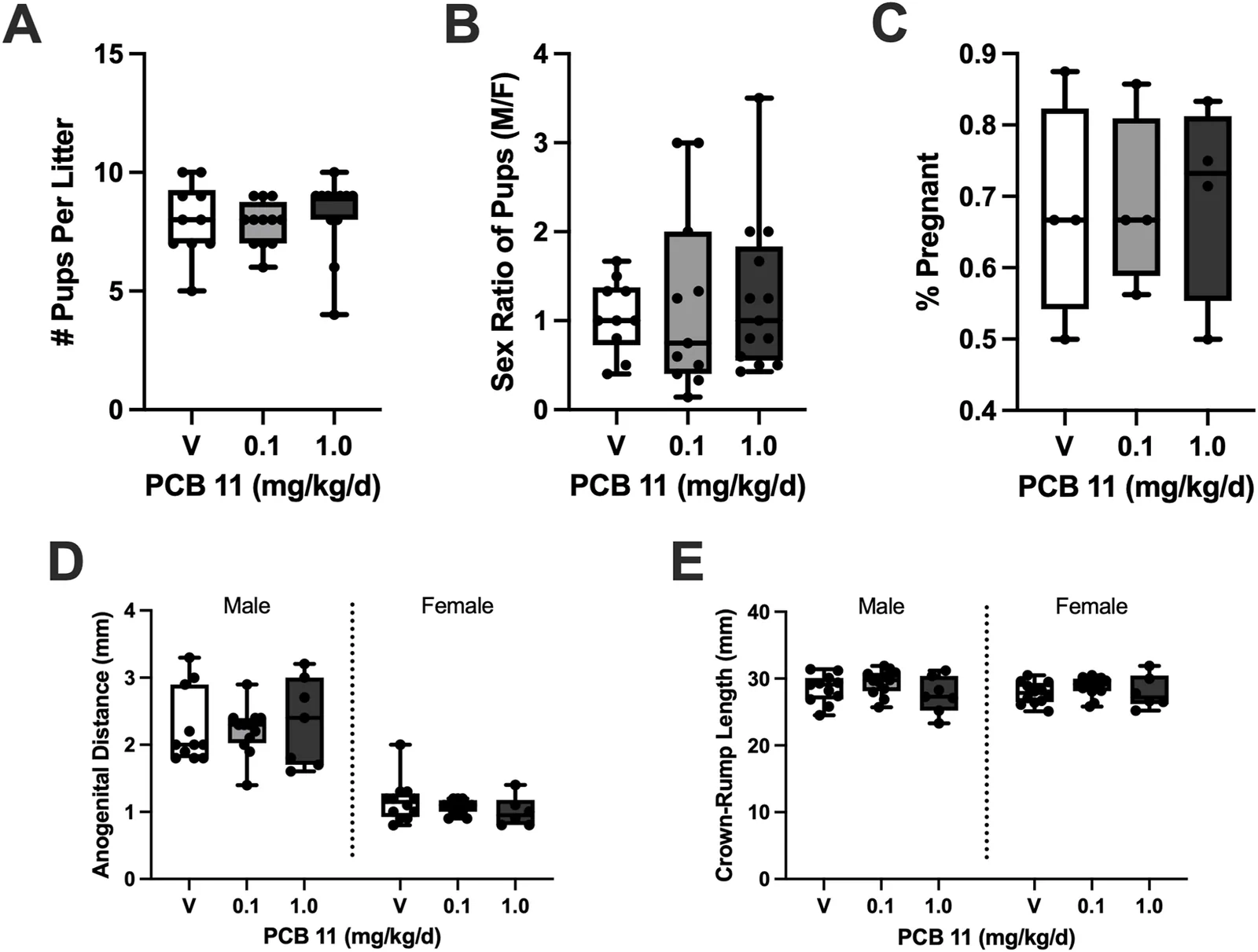
PCB 11 did not cause overt maternal or fetal toxicity. **(A)** The number of pups born per litter in each dose group (n = 10–12 litters per group); **(B)** sex ratio in each litter within each dose group presented as number of males/number of females (n = 10–12 litters per group); **(C)** percent of viable pregnancies for each cohort (n = 4 cohorts per group, 6–16 litters per cohort); **(D)** anogenital distance (n = 7–12 litters per sex per group; 3–4 pups averaged per sex per litter); and **(E)** crown-rump length (n = 7–12 litters per sex per group; 3-4 pups averaged per sex per litter). Data are presented as box and whisker plots in which the box indicates the 25th (lower) to 75th (upper) quartiles; the middle horizontal bar, the median; whiskers, the minimum and maximum values; and dots, the individual values. **(A,C)** Data were analyzed using one-way ANOVA or **(B,D)** two-way ANOVA with dose and sex as variables with *post hoc* Dunnet’s test.

To assess whether exposure to PCB 11 in the maternal diet starting 14 days prior to conception and continuing throughout gestation and lactation interfered with maternal health or general pup development, we measured the weight of dams and pups throughout the exposure period. There were no significant differences in dam weight between groups, as indicated by a mixed-effects analysis (Figure 1A). Similarly, PCB 11 had no significant effect on the weight of male or female pups from the time of birth until euthanasia at P21, as measured within sex using a mixed-effects analysis (Figures 1B,C). To further evaluate maternal and fetal toxicity, we assessed the total number of pups per litter (Figure 2A), sex ratio of pups in each litter (Figure 2B), the percent of viable pregnancies for each cohort (Figure 2C), anogenital distance (AGD) within each sex (Figure 2D), and crown-rump length (CRL) of pups (Figure 2E). No significant effects of exposure to PCB 11 were found for any of these measures, as indicated by one-way ANOVA for litter size, sex ratio, and percent pregnant (Figures 2A–C) or two-way ANOVA (p > 0.05) for AGD and CRL with dose and sex as variables (Figures 2D,E).

### 3.2 Developmental exposure to PCB 11 altered dendritic arborization in a dose-, brain region-, and sex-dependent manner

**FIGURE 3:**
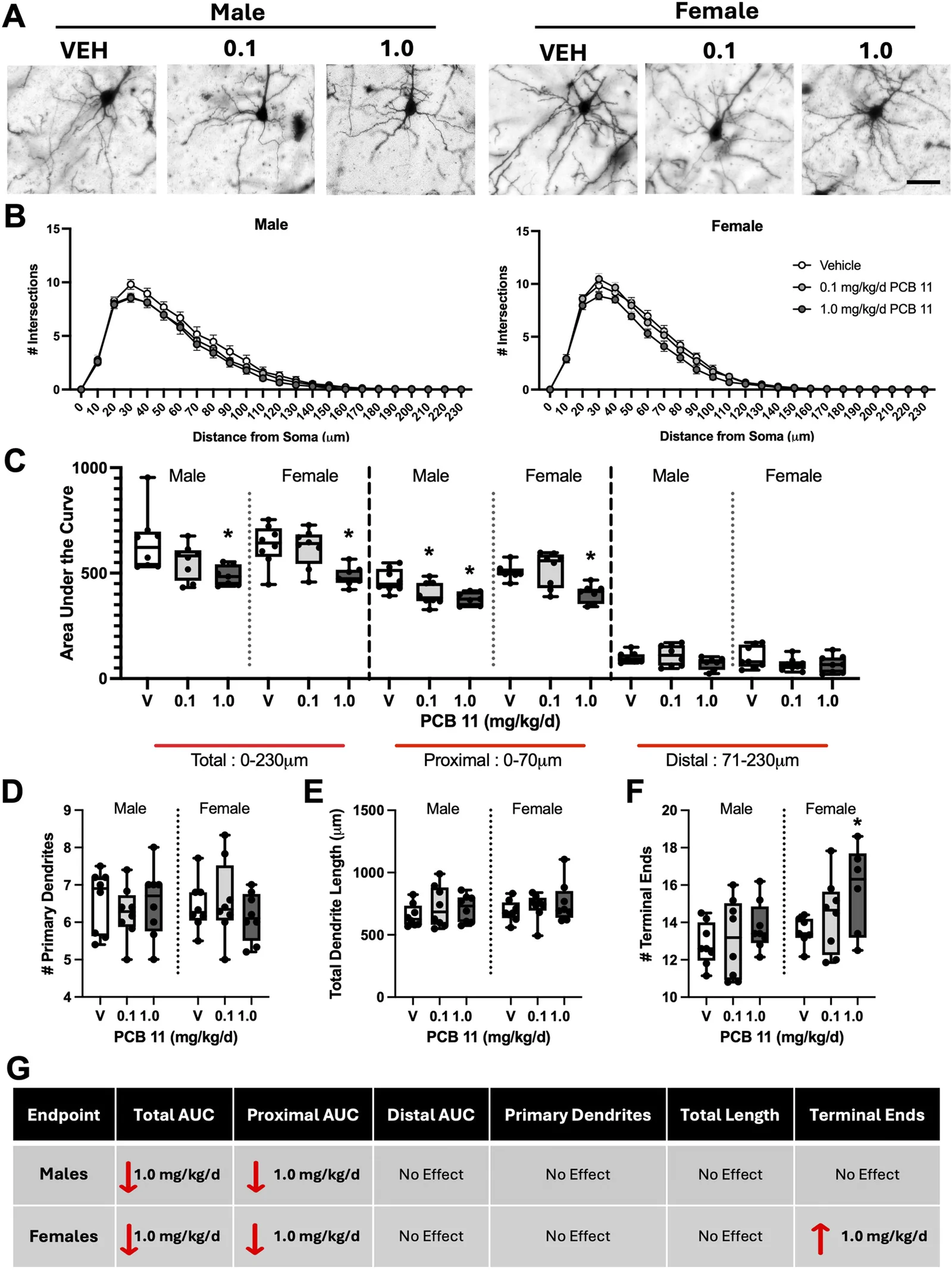
PCB 11 altered dendritic arborization of pyramidal neurons in layer V-VI of the mouse neocortex at P21. **(A)** Representative images of cortical neurons from PCB 11 and vehicle (V)-exposed male and female mice. The dendritic arbors of Golgi-stained neurons were traced from images obtained using a spinning disc confocal microscope (VT-1000, Leica, Solms, Germany) at ×40 magnification (scale bar = 50 μm) using Neurolucida software (version 11, MBF Bioscience, Williston, VT). **(B)** Dendritic complexity of individual neurons was quantified using Sholl analysis to measure the total number of dendritic intersections with each Sholl ring. **(C)** The total area under the curve (AUC) (0–230 μm), the proximal AUC (0–70 μm), and the distal AUC (71–230 μm) of the Sholl plot was quantified for male and female offspring independently. Tracings were also used to quantify **(D)** the total number of primary dendrites, **(E)** the total dendrite length per neuron (μm), and **(F)** the total number of dendritic terminals. **(G)** A summary table of dendritic alterations following PCB 11 exposure. Data are presented as box and whisker plots in which the box indicates the 25th (lower) to 75th (upper) quartiles; the middle horizontal bar, the median; whiskers, the minimum and maximum values; and dots, the individual values (n = 7–8 animals per group with 5–6 neurons analyzed per animal). Data were analyzed using mixed effects model. *P < 0.05 compared to sex-matched vehicle control.

To determine whether developmental exposure to PCB 11 altered dendritic growth *in vivo,* brains from P21 mouse pups exposed to PCB 11 in the maternal diet throughout gestation and lactation were Golgi stained to visualize basilar dendritic arbors of individual pyramidal neurons in layers V/VI of the cortex and in the hippocampus. The dendritic arbors of individual Golgi-stained neurons were analyzed by Sholl analysis (Figure 3; Supplementary Figure S2).

In male mouse pups, PCB 11 reduced dendritic arborization in cortical pyramidal neurons (Figure 3A). More specifically, the Sholl plots generated for male pups exposed to PCB 11 at 1.0 mg/kg/day in the maternal diet showed a significantly decreased total area under the curve (AUC) in the Sholl plot (F_(2, 20)_ = 4.716; p = 0.0210) relative to vehicle controls (Figure 3B). This decrease in dendritic complexity was driven by significantly decreased AUC in the proximal portion of the Sholl plot (0–70 μm from the soma) (F_(2, 20)_ = 6.156; p = 0.0062), with no PCB effects observed in the distal portion (71–230 μm) (Figure 3C). In male pups in the 0.1 mg/kg/day dose group, there were no significant PCB effects on the total AUC; however, the proximal AUC (F_(2, 20)_ = 6.156, p = 0.0347) was significantly reduced relative to vehicle controls. Other dendritic measures, including the number of primary dendrites (Figure 3D), total dendritic length (Figure 3E), number of terminal ends (Figure 3F), mean dendritic length (Supplementary Figure S1A), and number of nodes (Supplementary Figure S1B), were not significantly altered by exposure to PCB 11 (Figure 3G).

In female mouse pups, exposure to PCB 11 at 1.0 mg/kg/day similarly reduced the total AUC of the Sholl plot for cortical pyramidal neurons (F_(2, 20)_ = 7.149; p = 0.0048), again driven by changes in the proximal portion (F_(2, 19)_ = 7.761; p = 0.0099) but not the distal portion of the Sholl plot (Figure 3C). Unlike males, however, females exposed to PCB 11 exhibited a significant increase in the number of terminal ends (Figure 3F; F_(2, 20)_ = 3.516; p = 0.0491) while the number of primary dendrites (Figure 3D), total dendritic length (Figure 3E), mean dendritic length (Supplementary Figure S1A), and number of nodes (Supplementary Figure S1B) remained unchanged. In addition, PCB 11 had no significant effect on the area of the cell body (μm^2^) of cortical pyramidal neurons of either males or females (Supplementary Figure S1C).

PCB 11 had more subtle effects on hippocampal pyramidal neurons. In male mouse pups, there were no significant group differences in any of the parameters of dendritic arborization that were measured in hippocampal pyramidal neurons (Supplementary Figure S2). However, in female mouse pups in the 0.1 mg/kg/day dose group, PCB 11 significantly increased dendritic arborization compared to vehicle, as indicated by a significant increase in the proximal AUC (0–70 μm) (Supplementary Figure S2B; F (2, 21) = 3.961; p = 0.0330), but not the distal AUC. Effects of PCB 11 were not noted for the other measures of dendritic arborization (Supplementary Figure S2).

### 3.3 PCB 11 did not significantly alter apoptosis in the developing brain

**FIGURE 4:**
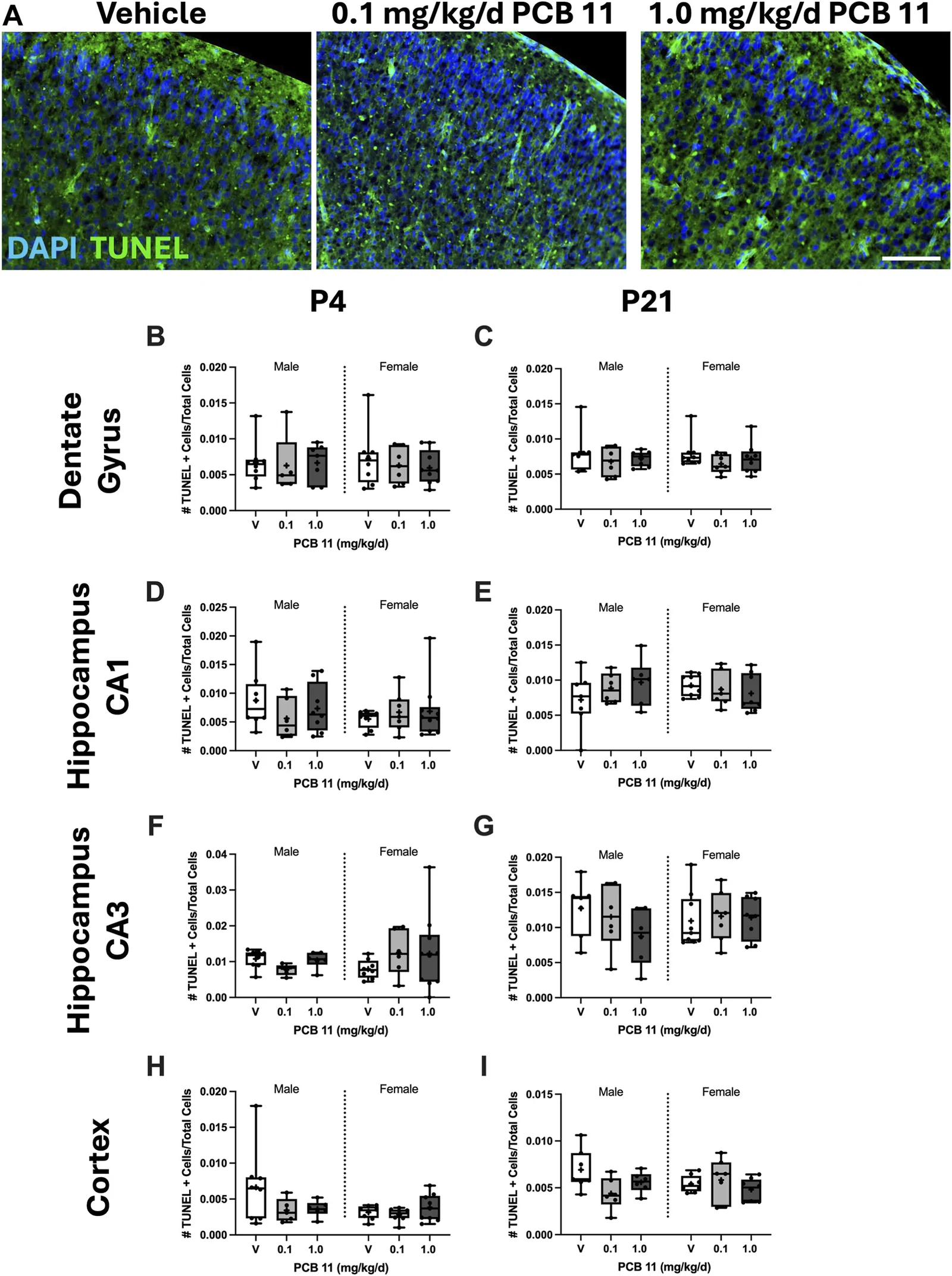
PCB 11 had no significant effect on apoptosis in the developing brain. TUNEL staining was used to measure apoptosis in brain tissue collected at P4 and P21. **(A)** Representative photomicrographs of TUNEL staining (green) in the lateral portion of the somatosensory cortex of vehicle and PCB 11-exposed female mice collected at P4; sections were counterstained with DAPI (blue) to identify nuclei (bar = 75 µm). Data are presented for TUNEL staining in the dentate gyrus **(B,C)**, CA1 region of the hippocampus **(D,E)**, CA3 region of the hippocampus **(F,G)**, and the somatosensory cortex **(H,I)**. The total number of TUNEL-positive cells was measured and normalized to the number of cell nuclei in the region of interest. Data are presented as box and whisker plots in which the box indicates the 25th (lower) to 75th (upper) quartiles; the middle horizontal bar, the median; whiskers, the minimum and maximum values; and dots, the individual values (n = 7–8 animals per group). Data were analyzed using two-way ANOVA with dose and sex as variables with *post hoc* Dunnet’s test, *P < 0.05 compared to sex-matched vehicle control.

**FIGURE 5:**
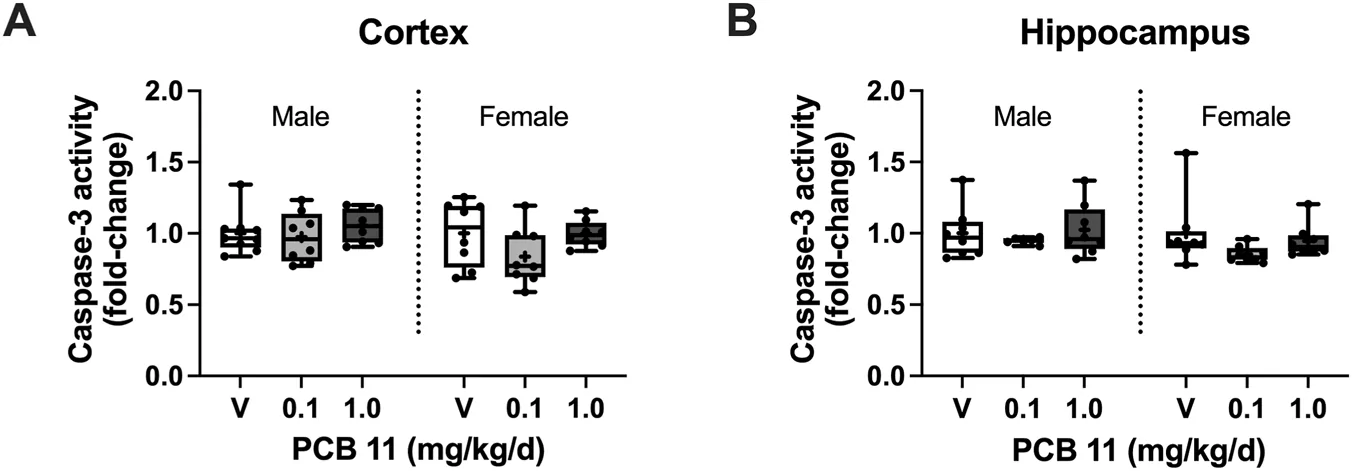
PCB 11 had no significant effect on brain region-specific caspase-3 activity at P4. A fluorometric assay was used to measure caspase-3 activity in the **(A)** cortex and **(B)** hippocampus. Data are presented as fold-change from control (vehicle, V) using box and whisker plots in which the box indicates the 25th (lower) to 75th (upper) quartiles; the middle horizontal bar, the median; whiskers, the minimum and maximum values; and dots, the individual values (n = 6–8 animals per group). Data were analyzed using two-way ANOVA with dose and sex as variables with *post hoc* Dunnet’s test, *P < 0.05 compared to sex-matched vehicle control.

To determine whether developmental exposure to PCB 11 altered apoptosis, we assessed apoptosis at P4 and P21 using TUNEL staining to detect DNA fragmentation (Figure 4). A two-way ANOVA with sex and dose as factors revealed no significant effects of PCB 11 on apoptosis at either timepoint in males or females across the brain regions assessed (dentate gyrus, hippocampal CA1 and CA3). Consistent with these results, quantification of caspase-3/7 activity, a key marker of apoptosis (Lamkanfi and Kanneganti, 2010) revealed no significant effect of PCB 11 in the cortex (Figure 5A) or hippocampus (Figure 5B) at P4 as indicated by two-way ANOVA with sex and dose as variables. Power analyses based on preliminary data (Pessah et al., 2016) indicate that the present design (6–8 animals per group per timepoint) provides approximately 80%–85% power to detect large effects (e.g., Cohen’s f = 0.4), and moderate power (∼65–70%) to detect medium-sized effects, under two-sided testing at α = 0.05. Thus, while smaller effects may not be reliably detected, this study is sufficiently powered to identify biologically meaningful differences, and the absence of significant differences suggests that there are no biologically meaningful alterations to apoptosis.

### 3.4 PCB 11 modulated astrocyte, but not microglial, reactivity in the developing brain

**FIGURE 6:**
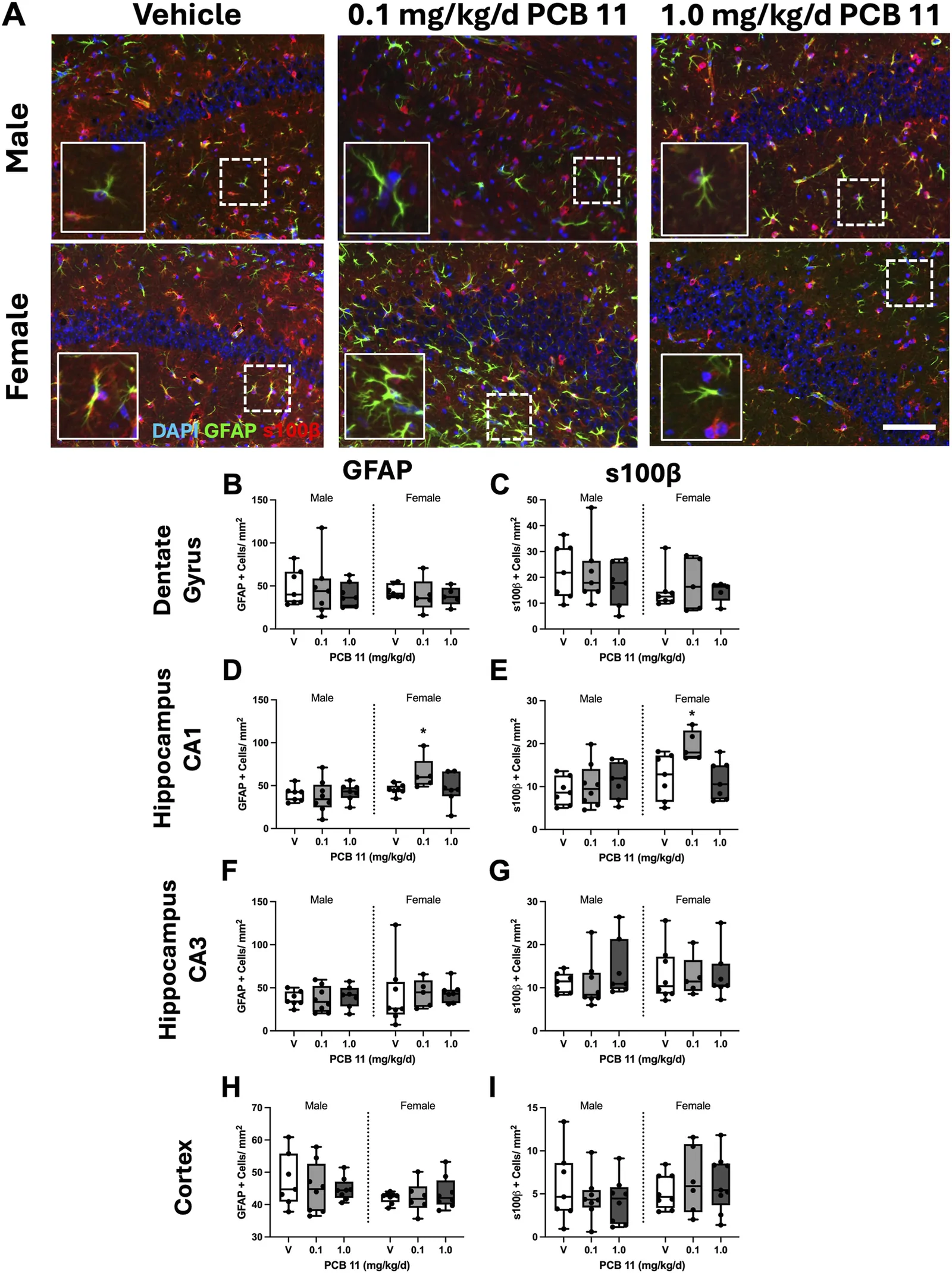
PCB 11 increased astrogliosis at P21 in a sex- and brain region-dependent manner. **(A)** Representative photomicrographs of GFAP (green) and s100β (red) immunoreactivity in the CA1 region of the hippocampus of vehicle and PCB 11-exposed male and female mice (bar = 100 μm). Sections were counterstained with DAPI (blue) to identify cell nuclei. The number of GFAP **(B,D,F,H)** and s100β **(C,E,G,I)** immunopositive cells per mm^2^ was quantified in the dentate gyrus **(B,C)**, the CA1 of the hippocampus **(D,E)**, the CA3 of the hippocampus **(F,G)**, and the somatosensory cortex **(H,I)**. Data are presented as box and whisker plots in which the box indicates the 25th (lower) to 75th (upper) quartiles; the middle horizontal bar, the median; whiskers, the minimum and maximum values; and dots, the individual values (n = 7–8 animals per group). Data were analyzed using two-way ANOVA with dose and sex as variables with *post hoc* Dunnet’s test, *P < 0.05 compared to sex-matched vehicle control.

Changes in the number and/or phenotype of astrocytes and microglia have been implicated in the etiology of neurodevelopmental disorders (Reemst et al., 2016; Xiong et al., 2023). As a first step in determining whether developmental exposure to PCB 11 modulates glial phenotypes, brain sections isolated from male and female pups at P21 were co-immunostained for GFAP and s100β to identify homeostatic astrocytes and radial glia vs. reactive and proliferative astrocytes, respectively (Yang and Wang, 2015; Zhang et al., 2019) (Figure 6). GFAP and s100β immunoreactivity were quantified in the dentate gyrus (Figures 6B,C), CA1 (Figures 6D,E) and CA3 of the hippocampus (Figures 6F,G), and the somatosensory cortex (Figures 6H,I). PCB 11 increased astrocytic activation in a dose-, sex-, and region-dependent manner (Figure 6A). Specifically, female mouse pups exposed to 0.1 mg/kg/d PCB 11 in the maternal diet exhibited an increased number of GFAP immunopositive cells (Figure 6D; F_(2, 16)_ = 3.898; p = 0.0418) and more s100β-positive cells (Figure 6E; F_(2, 16)_ = 5.116; p = 0.0192) in the CA1 region of the hippocampus, as measured by one-way ANOVA within sex with Dunnet’s *post hoc* compared to vehicle control. No other significant effects of PCB 11 were observed.

**FIGURE 7:**
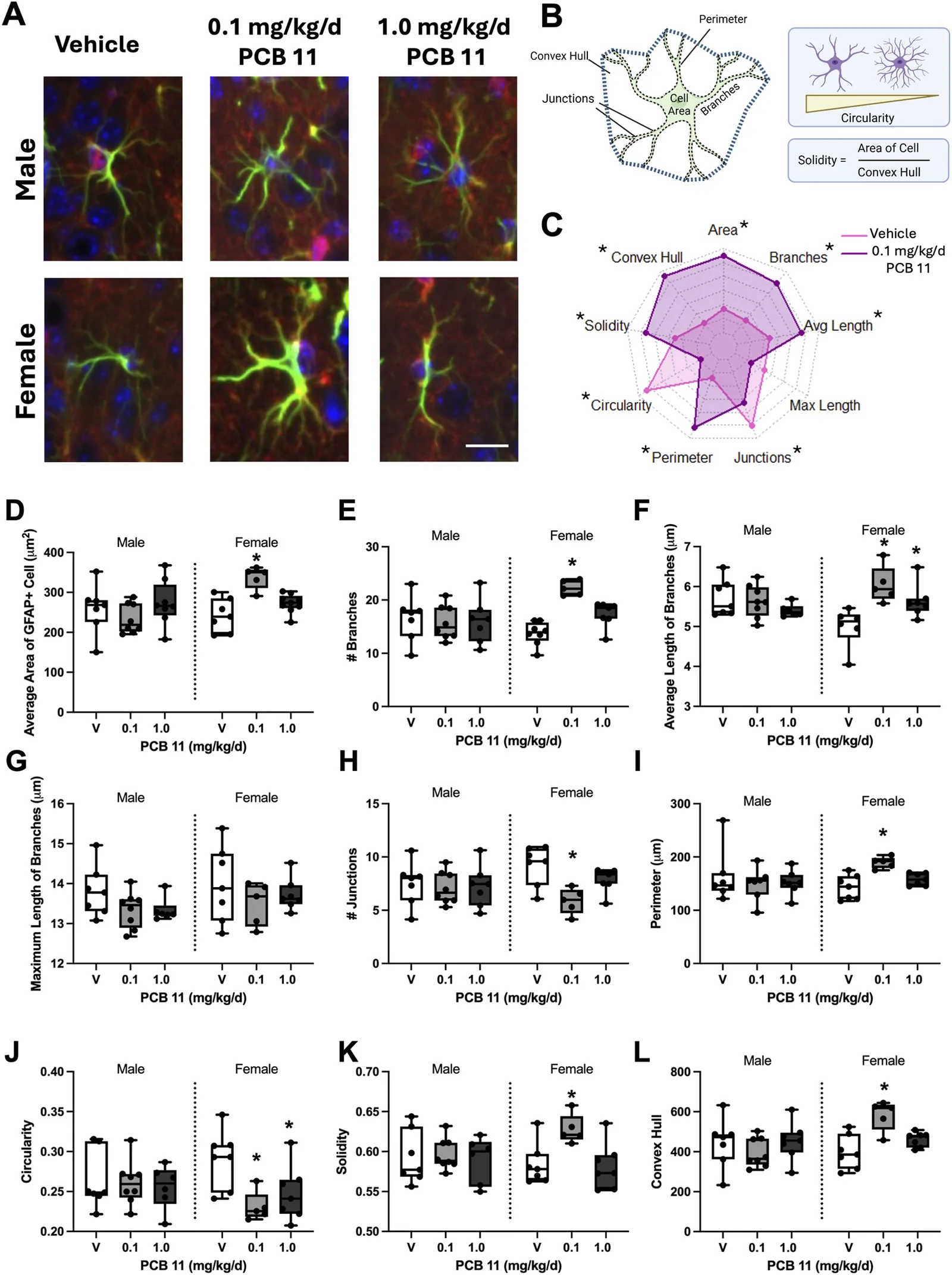
PCB 11 altered astrocyte morphology in the CA1 hippocampus of P21 female mice. **(A)** Representative photomicrographs of GFAP (green) and s100β (red) immunoreactivity in the CA1 hippocampus of vehicle and PCB 11-exposed male and female mice (bar = 5 μm). Sections were counterstained with DAPI (blue) to identify cell nuclei. **(B)** Schematic illustrating the morphological endpoints that were quantified. **(C)** A radial plot summary comparing vehicle control (light pink) and the 0.1 mg/kg/day (purple) female mice. Minimum and maximum values of the radial plot are one standard deviation away from the maximum and minimum values for each endpoint. Astrocyte morphology was assessed with respect to: **(D)** the average area of individual GFAP immunopositive cells, **(E)** total number of branches, **(F)** average length of branches, **(G)** maximum length of branches, **(H)** total number of junctions, **(I)** cell perimeter, **(J)** circularity, **(K)** solidity, and **(L)** convex hull area. Data are presented as box and whisker plots in which the box indicates the 25th (lower) to 75th (upper) quartiles; the middle horizontal bar, the median; whiskers, the minimum and maximum values; and dots, the individual values (n = 7–8 animals per group, 152–460 GFAP positive cells averaged per animal). Data were analyzed using two-way ANOVA with dose and sex as variables with *post hoc* Dunnet’s test, *P < 0.05 compared to sex-matched vehicle control.

PCB 11 altered astrocytic morphology in a dose-, brain-region-, and sex-dependent manner (Figures 7A–C). Compared to sex-matched vehicle controls, GFAP-immunopositive cells in the CA1 hippocampus of female mouse pups exposed to 0.1 mg/kg/d PCB 11 in the maternal diet exhibited increased cell area (Figure 7D), number of branches (Figure 7E), average length of branches (Figure 7F), perimeter (Figure 7I), solidity (Figure 7K), and convex hull (Figure 7L). In contrast, there was a reduction in the number of junctions (Figure 7H) and circularity (Figure 7J), while the maximum branch length was unchanged (Figure 7G). Statistical values are provided in Table 1. No significant effects of PCB 11 on astrocyte morphology were observed in other brain regions in female mouse pups or in any brain region in male mouse pups (data not shown).

**TABLE 1:** Effects of PCB 11 on astrocyte morphology in the CA1 of the hippocampus in male and female juvenile mice.

Morphological endpoints	Male		Female	
	0.1 mg/kg/day PCB11	1.0 mg/kg/day PCB 11	0.1 mg/kg/day PCB 11	1.0 mg/kg/day PCB 11
Cell area	p > 0.05	p > 0.05	**F (2, 38) = 2.183; p = 0.0019**	p > 0.05
# Branches	p > 0.05	p > 0.05	**F (2, 36) = 5.190; p < 0.0001**	p > 0.05
Avg length	p > 0.05	p > 0.05	**F (2, 33) = 4.511; p = 0.0005**	**F (2, 33) = 4.511; p = 0.0239**
Max length	p > 0.05	p > 0.05	p > 0.05	p > 0.05
# Junctions	p > 0.05	p > 0.05	**F (2, 35) = 4.198; p = 0.0042**	p > 0.05
Perimeter	p > 0.05	p > 0.05	**F (2, 34) = 3.521; p = 0.0222**	p > 0.05
Circularity	p > 0.05	p > 0.05	**F (2, 34) = 3.738; p = 0.0068**	**F (2, 34) = 3.738; p = 0.0371**
Solidity	p > 0.05	p > 0.05	**F (2, 34) = 3.882, p = 0.0202**	p > 0.05
Convex hull	p > 0.05	p > 0.05	**F (2, 35) = 6.544; p = 0.0023**	p > 0.05

Values in the table represent F values, including degrees of freedom, and the corresponding p-values following *posthoc* analysis. Bolded text indicate p < 0.05 (n = 7–8 animals per experimental condition).

To identify microglia reactivity, brain sections collected at P21 were immunostained for Iba1, which identifies microglia and macrophages, and CD68, which identifies phagocytic cells (Jurga et al., 2020) (Supplementary Figure S3). Iba1 and CD68 immunoreactivity were quantified in the dentate gyrus (Supplementary Figures S3B,C), CA1 (Supplementary Figures S3D,E) and CA3 of the hippocampus (Supplementary Figures S3F,G), and the somatosensory cortex (Supplementary Figures S3H,I). There were no significant effects of PCB 11 detected for either biomarker in any of these brain regions in either sex as indicated by one-way ANOVA within each sex.

### 3.5 PCB 11 disrupted neurogenesis in the developing brain

Altered neurogenesis has been implicated in the etiology of neurodevelopmental disorders and has been observed in mouse models of NDD (Barón-Mendoza et al., 2024; Courchesne et al., 2019). To assess neurogenesis, brain sections collected at P4 and P21 from male and female mouse pups exposed to vehicle or PCB 11 in the maternal diet were immunostained for Ki-67 to identify proliferating cells (Gerdes et al., 1984), DCX to label migrating neurons (Gleeson et al., 1999), and NeuN, a biomarker of mature neurons (Wolf et al., 1996), as previously described (Patten et al., 2020). At P4, no group differences were observed in Ki67, DCX, or NeuN immunostaining in the SGZ or GCL of the dentate gyrus (Supplementary Figure S3) as measured by a two-way ANOVA using sex and dose as variables with *post hoc* Tukey’s test.

**FIGURE 8:**
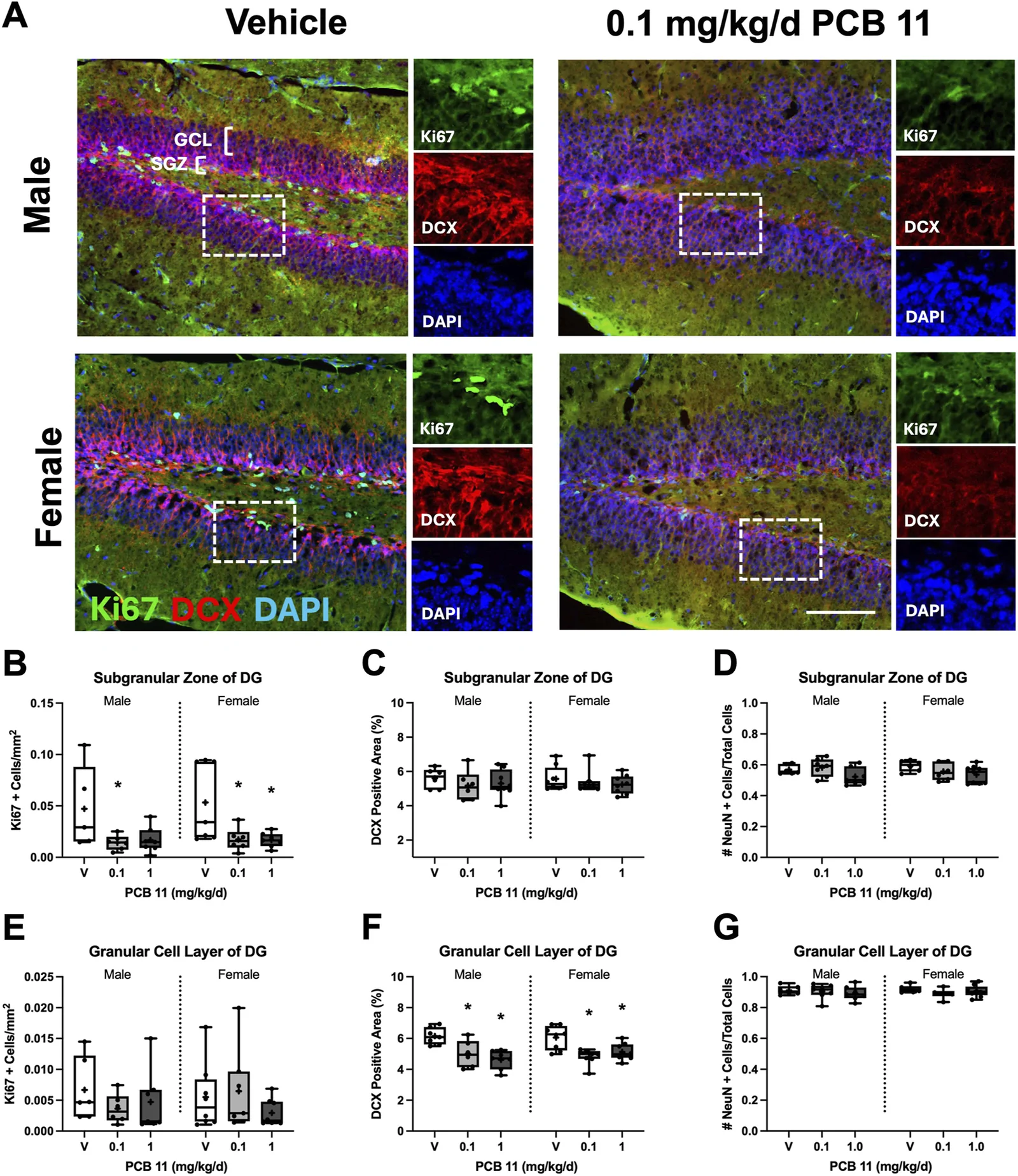
PCB 11 dysregulated neurogenesis at P21. **(A)** Representative photomicrographs of Ki67 (green) and doublecortin (DCX, red) immunoreactivity in the subgranular zone (SGZ) and granular cell layer (GCL) of dentate gyrus in vehicle and PCB-11-exposed male and female mice (bar = 100 μm). Sections were counterstained with DAPI (blue) to identify cell nuclei. The number of Ki67 immunopositive cells per mm^2^ **(B,E)**, percent area immunopositive for DCX **(C,F)**, and number of cells immunopositive for NeuN over the total number of DAPI positive cells **(D,G)** were quantified in the SGZ **(B–D)** and GCL **(E–G)**. Data are presented as box and whisker plots in which the box indicates the 25th (lower) to 75th (upper) quartiles; the middle horizontal bar, the median; whiskers, the minimum and maximum values; and dots, the individual values (n = 6–8 animals per group). Data were analyzed using two-way ANOVA with sex and dose as variables, with *post hoc* Tukey’s test, *P < 0.05 compared to vehicle control.

In contrast, immunohistochemical staining of brain sections collected at P21 revealed significant, sex-specific effects of PCB 11 on biomarkers of neurogenesis (Figure 8A). In male mouse pups exposed to PCB 11 at 0.1 mg/kg/day in the maternal diet the density of Ki67-positive cells in the SGZ of the dentate gyrus was significantly reduced relative to vehicle controls (Figure 8B; F_(2, 32)_ = 8.555; p = 0.4994) as indicated by a two-way ANOVA using sex and dose as variables with *post hoc* Tukey’s test. Similarly, female pups exposed to PCB 11 at 0.1 and 1.0 mg/kg/day had significantly lower densities of Ki67-positive cells in the SGZ (Figure 8B; F_(2, 32)_ = 8.555; p = 0.0238 and p = 0.0164, respectively) than their vehicle counterparts There were no significant differences observed in density of DCX-positive stain (Figure 8C) or total NeuN-positive cells (Figure 8D) in the SGZ. Male mouse pups in the 0.1 and 1.0 mg/kg/day PCB 11 dose groups also exhibited a significant reduction in the percent area immunopositive for DCX in the GCL (Figure 8F; F_(2, 37)_ = 17.18; p = 0081 and p = 0.0002, respectively) as indicated by two-way ANOVA using sex and dose as variables with *post hoc* Tukey’s test. Female mouse pups exposed to PCB 11 at 0.1 and 1.0 mg/kg/day in the maternal diet showed a similar decrease in DCX immunostaining in the GCL (Figure 8F; F_(2, 37)_ = 17.18; p = 0.0017 and p = 0.0182, respectively). There were no significant differences in the density of Ki67-positive cells (Figure 8E) or total NeuN-positive cells (Figure 8G) in the GCL.

## 4 Discussion

The objective of this study was to test the hypothesis that gestational and lactational exposure to PCB 11 in the maternal diet affects dendritic morphogenesis and other neurodevelopmental processes relevant to NDD, including autism, in the postnatal mouse brain. Our findings support this hypothesis. Specifically, we observed that PCB 11 significantly altered dendritic arborization, disrupted neurogenesis, and increased astrocyte reactivity in juvenile mice in a dose-, brain region-, and sex-dependent manner. While interactions between PCB exposure and environmental factors such as cage plastics or corncob bedding cannot be ruled out, all animals were maintained under identical housing conditions. Therefore, these factors are unlikely to explain the observed differences between PCB-exposed and vehicle control groups. Importantly, these changes were not attributable to general maternal toxicity or adverse effects on general pup growth and development, as evidenced by the absence of PCB 11 effects on dam or pup weight throughout the exposure period, the size or sex ratio of litters, and general measures of pup development, specifically, the crown-rump length and anogenital distance in male or female pups.

Modulation of the spatiotemporal patterns of dendritic morphogenesis following developmental exposure to PCBs has been reported in a number of studies (Klocke et al., 2019), and aberrations in dendritic number, length, branching, and/or timing of dendritic outgrowth are thought to contribute to the clinical symptoms observed in human neuropsychiatric disorders (Supekar et al., 2013; Copf, 2016; Lamanna and Meldolesi, 2024). In this study, we analyzed the pyramidal CA1 hippocampal neurons and layer V/VI pyramidal somatosensory cortical neurons because changes in the dendritic morphology of these neuronal cell types have been observed in human NDD, including autism (Keown et al., 2013; Coskun et al., 2013; Raymond et al., 1996; Forrest et al., 2018). We differentiated between PCB 11 effects on the proximal versus distal basal dendrites to gain insight regarding sensitive windows of exposure and functional impacts of PCB 11 dendritic effects. The observation that PCB 11 had greater impact on proximal dendrites, which are formed before distal dendrites, suggests that PCB 11 is interfering with early stages of dendritogenesis and not modifying later stages of dendritic arbor refinement. Proximal vs. distal dendrites differentially contribute to the biomechanics of neuronal activity. While distal excitation lowers the local spike threshold with little effect on the peak responses in the soma of the neuron, more proximal excitation lowers the threshold and substantially increases somatic response, leading to a larger effect on the cell’s firing rate (Behabadi et al., 2012).

Previous *in vitro* studies demonstrated that PCB 11 enhanced dendritic arborization in rat hippocampal and cortical pyramidal neurons grown in primary neuron-astrocyte co-cultures (Sethi et al., 2017a; Sethi et al., 2018). The dendrite-promoting effects of PCB 11 were observed in female but not male hippocampal neuron-glia co-cultures; whereas in cortical neuron-glia co-cultures, PCB 11 enhanced dendritic arborization in male but not female neurons (Sethi et al., 2017b). *In vivo*, we found that developmental exposure to PCB 11 at 0.1 mg/kg/day in the maternal diet similarly enhanced dendritic growth of pyramidal neurons in the female but not male CA1 hippocampus, although the dendrite-promoting effect *in vivo* was relatively subtle, manifesting as a significant increase in the proximal AUC of the Sholl plot. However, in pyramidal neurons in layer V/VI of the somatosensory cortex, the complexity of the dendritic arbor was decreased in males in the 0.1 and 1.0 mg/kg/day PCB 11 dose groups and in females in the 1.0 mg/kg/day PCB 11 dose group. The reason for the discrepancies between the previous *in vitro* and current *in vivo* findings with respect to PCB 11 effects on the dendritic morphogenesis of cortical neurons are not known. One possibility are differences in PCB 11 concentrations in cellular targets. Unfortunately, we do not have data regarding PCB 11 levels in the fetus during the exposure period, thus, we cannot derive an approximate concentration in the developing brain to compare with PCB 11 concentrations observed to interfere with dendritogenesis *in vitro*. Another possible explanation is the absence of microglia and oligodendrocytes in the *in vitro* models as these cell types are implicated in the refinement of dendritic arborization *in vivo* (Ji et al., 2013). Additionally, *in vitro* cultures included neurons from the entire cortex*,* whereas *in vivo* analyses targeted specific neuronal subtypes (e.g., layer V/VI cortical or CA1 hippocampal pyramidal neurons). Alternatively, and/or in addition, the differences in dendritic arborization of cortical neurons *in vitro* vs. *in vivo* may reflect differences in the maturational stage of the cortical neurons at the time of analysis, which is known to influence dendritic arborization and response to extrinsic cues, including PCBs (Keil et al., 2017; Yang et al., 2009; Pessah et al., 2019).

Our findings are also in contrast with a previous study of dendritic arborization in the hippocampus and cortex of P27-P31 mouse pups exposed to the MARBLES PCB mixture in the maternal diet at 0.1, 1.0, or 6.0 mg/kg/day throughout gestation and lactation (Keil Stietz et al., 2021). PCB 11 comprised 24.3% of the MARBLES mix, which was formulated to mimic the relative proportions of the twelve most abundant congeners found in the serum of pregnant women at increased risk for having a child with a neurodevelopmental disorder (Sethi et al., 2019). The MARBLES PCB mixture was reported to have no effect on the dendritic arborization of hippocampal neurons in WT mice of either sex but significantly increased dendritic arborization of cortical neurons in males in the 6 mg/kg/day MARBLES dose group. The difference in dendritic outcomes between mice exposed to the MARBLES mix vs. PCB 11 alone is likely attributable to the presence of additional PCB congeners in the MARBLES mixture, which could mask or counteract the effects of PCB 11 despite its high relative abundance in the MARBLES mixture. Another factor that may contribute to the discrepant findings is the age of the mice at the time of analysis.

We did not observe any effects of PCB 11 on apoptosis in either the hippocampus or cortex at P4 or P21 as determined using two different methods to quantify apoptosis. Previous studies have reported inconsistent effects of PCB congeners on neuronal apoptosis, suggesting that individual congeners may differentially affect this neurodevelopmental process. For example, in a study of primary rat cultures, PCB 47, a non-dioxin like (NDL) congener, increased neuronal apoptosis, but neither PCB 77, a dioxin-like (DL) congener nor PCB 104, a NDL congener, exhibited pro-apoptotic activity (Howard et al., 2003). Whether an individual PCB congener enhances apoptosis may be determined by its activity at the ryanodine receptor (RyR), activation of which can trigger calcium-dependent pathways that promote neuronal apoptosis (Berridge et al., 2000; Howard et al., 2003). Both PCB 77 and PCB 104 were found to have negligible activity at the RyR, whereas PCB 47 had significant activation of the receptor (Pessah et al., 2006). PCB 11 has been demonstrated to have negligible activity at the RyR (Sethi et al., 2018), consistent with our observation that developmental exposure to PCB 11 did not cause significant dysregulation of apoptosis in the P4 or P21 mouse hippocampus or cortex.

An interesting finding of this study was that developmental PCB 11 exposure increased some measures of astrocytic reactivity but had no effect on microglial reactivity. Changes in the number and/or reactivity of these neuroimmune cells have been linked to cognitive deficits and increased risk of NDD, including autism (Hanamsagar and Bilbo, 2017; Xiong et al., 2023). Astrocytes regulate neuroimmune responses in the developing brain; for example, astrocytes respond to immune challenge by becoming reactive, and by releasing pro-inflammatory factors that can have either beneficial or detrimental effects, depending on the physiological context (Liddelow and Barres, 2017; Escartin et al., 2021). Reactive astrocytes exhibit increased expression of GFAP and/or s100β (Yang and Wang, 2015; Zhang et al., 2019), and their morphology changes. The morphological complexity of astrocytes is a central feature of glial cell biology and a key structural basis for multicellular spatial organization and physiological interactions within the central nervous system (Baldwin et al., 2024). In general, reactive astrocytes have more extensive and complex morphology compared to resting astrocytes (Delgado-García et al., 2024). Chronic astrocytic reactivity, as determined by increased levels of GFAP, abnormal levels of the astrocyte biomarkers APQ4 and CX43, and disruption of astrocytic function, including regulation of neurotransmitter homeostasis and ion channels, has been observed in NDD, including autism (Molofsky et al., 2012; Xiong et al., 2023).

Our observation that developmental exposure to PCB 11 at 0.1 mg/kg/day increased astrocyte reactivity in the CA1 subregion of the hippocampus of P21 female mice aligns with previous *in vivo* observations of offspring of rat dams exposed to Aroclor 1254 at 5.0 or 25.0 mg/kg/day from gestational days 10–16. Significantly increased GFAP expression was observed in the lateral olfactory tract and cerebellum of P21 and P90 rat pups (Morse et al., 1996). In an independent study of Long-Evans rat pups exposed to the Fox River PCB mixture at 3.0, 6.0, or 18.0 mg/kg/day during gestation and lactation (Miller et al., 2010), GFAP immunoreactivity was decreased in males but significantly increased in females at P21, suggesting a sex-dependent astrocytic response. In our study, changes in astrocyte immunoreactivity paralleled the dose- and sex-dependent alterations observed in hippocampal dendritic morphology. Previous work demonstrated that increased astrocyte activation, as determined by increased GFAP biomarker expression, can upregulate astrocyte-derived phospholipase D1 (Lee et al., 2000), which has been implicated in promoting dendritic arborization (Zhu et al., 2016). These findings suggest that heightened astrocyte reactivity may have contributed to the increased dendritic complexity observed in the hippocampus of PCB 11-exposed females. But the sex- and dose-dependent effects of PCB 11 on astrocyte reactivity do not align well with PCB 11 effects on dendritic arborization observed in the cortex, arguing against a primary role for astrocytic reactivity in the cortical dendritic alterations observed in PCB 11-exposed pups.

Microglia are involved in the innate immune response in the brain, producing inflammatory cytokines as a first line of defense in response to injury, inflammation, and disease (Schafer and Stevens, 2015; Bronzuoli et al., 2018; Badimon et al., 2020). Microglia are also involved in neural circuit development via modulation of synaptogenesis and synaptic pruning (Paolicelli et al., 2011; Badimon et al., 2020). However, in contrast to astrocytes, we observed no effects of PCB 11 on microglial activation as defined by Iba1 and CD68 biomarker expression. Our finding is consistent with another study of rats exposed to a mixture of Aroclors 1242, 1248, and 1254 throughout gestation and lactation that reported no significant changes in microglial morphology, another measure of activation, or expression of genes linked to microglial reactivity (Walker et al., 2024). Collectively, the available data suggest that if PCB 11 exposure triggers microglial activation in the developing brain, this response does not persist at P21. The parsimonious conclusion is that PCB 11-induced changes in dendritic complexity are unlikely to be driven by microglial-dependent mechanisms.

The development of functional neural circuits also depends on precise regulation of the timing and extent of neurogenesis in the developing brain (Kriegstein, 2005; Silbereis et al., 2016), and spatiotemporal disruptions of this process are associated with a number of NDD (Ciptasari and van Bokhoven, 2020; Fan and Pang, 2017). We observed that developmental exposure to 0.1 or 1.0 mg/kg/day PCB 11 reduced the number of mitotic cells and migrating neurons in the dentate gyrus of both male and female pups at P21. Prior research similarly reported that neurogenesis is sensitive to PCBs (Naveau et al., 2014). For example, one study found increased BrdU labeling, a biomarker for active DNA synthesis and cell proliferation, in the ventromedial nucleus of P1 Sprague-Dawley rats exposed to 1.0 mg/kg/day of Aroclor 1221 via injection of the pregnant dam at gestational day 12, 14, or 16 (Hernandez Scudder et al., 2020). Shifts in the temporal patterns of neurogenesis have been connected to altered dendritic arborization (Gilbert and Man, 2017; Mottahedin et al., 2017). The PCB 11-induced changes in neurogenesis within the dentate gyrus at P21 paralleled decreased cortical dendritic arborization. Notably, the direction and magnitude of these two outcomes were aligned with consistent sex- and dose-dependent patterns. While this observation suggests the possibility that PCB 11-induced perturbations in neurogenesis contribute to alterations in dendritic arborization, further work will be required to determine whether these outcomes are causally linked.

The molecular mechanism(s) by which PCB 11 interferes with dendritic morphogenesis, astrocyte reactivity and/or neurogenesis remain to be determined as does the question of whether convergent mechanism(s) mediate PCB 11 effects on all three neurodevelopmental processes. Prior studies have identified RyR-dependent signaling, generation of reactive oxygen species (ROS), and cAMP response element binding protein (CREB)-dependent signaling as mechanisms by which NDL PCBs modulate the spatiotemporal patterns of dendritic arborization and/or apoptosis (Pessah et al., 2019; Klocke and Lein, 2020; Minuti et al., 2026). PCB 11 was reported to enhance dendritic growth in primary cortical neurons in neuron-astrocyte co-cultures via CREB-dependent mechanisms independent of effects on RyR activity (Sethi et al., 2018), but whether CREB-dependent mechanisms underlie the PCB 11 effects we observed *in vivo* has yet to be tested. CREB-dependent signaling is important in learning, memory, and synaptic plasticity (Bourtchuladze et al., 1994; Zheng et al., 2016), and genetic mutations in CREB signaling are associated with increased risk of multiple NDD, including autism (Sakamoto et al., 2011; Sweatt and Weeber, 2003; D’Andrea et al., 2015). Thus, if PCB 11 modulates neurodevelopment in the intact brain via CREB-dependent mechanisms, it would suggest the hypothesis that individuals who carry autism-associated mutations in CREB signaling may be more susceptible to the developmental neurotoxicity of PCB 11.

Numerous aspects of the experimental design of this study strengthen its translational relevance. First, there is evidence that PCB 11 is found in the human gestational environment (Sethi et al., 2019). Second, the dosing paradigm used in this study modelled human-relevant exposures to PCB 11 with respect to both route (Weitekamp et al., 2021; Sethi et al., 2019) and level of exposure (Wang et al., 2026). Third, the neurodevelopmental endpoints assessed, e.g., dendritic arborization, apoptosis, neuroimmune cell reactivity, and neurogenesis are cellular processes of neurodevelopment that are highly conserved across species and are consistently implicated in the etiopathogenesis of NDD, including autism (Courchesne et al., 2019; Al Dera, 2022).

Some observations from this study do not align with epidemiological patterns observed in autism. For example, in this study, female mice were more responsive to PCB 11 than males, whereas autism is more frequently diagnosed in males than females (Santos et al., 2022; Goodman et al., 2023). However, sex-differences in vulnerability to environmental toxins do not always correspond to disorder prevalence because the direction and magnitude of effects vary across toxicants, exposure windows, and the endpoint assessed (Torres-Rojas and Jones, 2018). Moreover, disorder prevalence may be influenced by factors independent of toxicant susceptibility, including the proposed female protective effect in autism or differences in symptom presentation that may contribute to reduced diagnosis in female patients (Lai and Szatmari, 2020; Ochoa-Lubinoff et al., 2023; Talukdar and Page, 2026). Further, we did not observe any alterations in apoptosis or microglial reactivity and morphology. Altered apoptosis has been implicated in autism; however, findings are inconsistent across cohorts and experimental models, with evidence supporting both increased and decreased apoptotic signaling, suggesting that altered apoptosis is not a universal feature of autism (Sheikh et al., 2010; Wei et al., 2014; Dong et al., 2018; Hess et al., 2021; Ming et al., 2022). Microglia have likewise been implicated in autism etiology, with altered activation states and immune signaling reported across multiple neurodevelopmental disorders (Szydlowska et al., 2026; Li et al., 2026). Some evidence suggests these changes may be specific to brain-region or developmental window (Ren et al., 2026); thus, the absence of detectable effects in the present study does not rule out PCB 11-induced alterations in microglial function in other brain regions or developmental windows.

A key question raised by these observations is whether the neurodevelopmental changes induced by developmental PCB 11 exposure translate into behavioral phenotypes relevant to autism and other neurodevelopmental disorders (NDDs) in animal models. Hallmarks of autism include early-life difficulties in social behavior and highly restrictive, repetitive behaviors, which are thought to reflect underlying impairments in social cognition, social perception, executive function, and perceptual and information-processing mechanisms. (Lai et al., 2014; Wang et al., 2023). This question is particularly relevant given evidence from both human and animal studies linking developmental PCB exposure to impairments in cognitive, attentional, and executive function domains commonly affected in autism and other NDDs (Berghuis et al., 2018; Mehri et al., 2021; Xu et al., 2023; Keil-Stietz and Lein, 2023; Wilson et al., 2024; Duque-Cartagena et al., 2024). Future studies that directly assess synaptic and circuit-level alterations, as well as studies examining PCB 11 effects in established models of NDDs, will be important for determining whether the cellular changes caused by developmental PCB 11 exposure scale to systems-level dysfunction, and whether PCB 11 interacts with genetic risk factors for NDD to influence NDD-relevant outcomes.

In summary, our findings suggest that developmental exposure to the LC-PCB congener, PCB 11, modulates critical neurodevelopmental processes *in vivo* that are of relevance to autism, further supporting the hypothesis that LC-PCBs in general, and PCB 11 in particular, represent an environmental risk factor for autism and NDD with shared phenotypes. Yet to be determined is whether the *in vivo* dendritic effects of PCB 11 are mediated by CREB-dependent signaling and whether the subtle, but significant, changes in cortical dendritogenesis, astrocyte activation, and neurogenesis translate to behavioral impairments of relevance to autism. Addressing these questions will be critical for understanding the risk that PCB 11 poses to the developing brain, and for identifying genetic susceptibilities that influence individual risk for PCB 11 developmental neurotoxicity.

## Data Availability

The datasets presented in this study can be found in online repositories. The names of the repository/repositories and accession number(s) can be found below: Dryad Data Repository at DOI: 10.5061/dryad.t76hdr8fb.
