# Supplementary material for: Neurodevelopmental outcomes relevant to autism in juvenile mice exposed to PCB 11 in the maternal diet throughout gestation and lactation
Source: Front Toxicol. 2026 Aug 12;8:1816944. doi: 10.3389/ftox.2026.1816944 (PMC13506163; doi:10.3389/ftox.2026.1816944)
Supplement: Supplementary file 1 [file DataSheet1.docx]

Neurodevelopmental Outcomes Relevant to Autism in Juvenile Mice Exposed to PCB 11 in the Maternal Diet Throughout Gestation and Lactation

Rebecca J. Wilson^1^, Harmanpreet K. Panesar^1^, Ilknur Dursun^1,2^, Rosalia Mendieta^1^, Peter M. Andrew^1^, Xueshu Li^3^, Hans-Joachim Lehmler^3^, and Pamela J. Lein^1*^

^1^ Department of Molecular Biosciences, University of California Davis, Davis, CA, USA

^2^ Istinye University, School of Medicine, Department of Physiology, Istanbul 34396, Turkey

^3^ Department of Occupational and Environmental Health, University of Iowa, Iowa City, IA, USA

*** Correspondence:**	Pamela J Lein
pjlein@ucdavis.edu

**Supplementary Files:**

- **Supplemental Figure 1.** *Additional parameters of dendritic arborization quantified in Golgi-stained cortical neurons at P21.*
- **Supplemental Figure 2**. *PCB 11 subtly altered the dendritic arborization of pyramidal neurons in the mouse hippocampus at P21.*
- **Supplementary Figure 3.** *PCB 11 did not significantly alter microglial number or phagocytic phenotype at P21.*
- **Supplemental Figure 3**. *PCB 11 did not dysregulate neurogenesis at postnatal (P) 4 in male or female weanlings.*

**SUPPLEMENTAL FIGURES**

**Supplemental Figure 1**. ***Additional parameters of dendritic arborization quantified in Golgi-stained cortical neurons at P21.*** Tracings of cortical neurons from PCB 11 and vehicle exposed male and female mice were analyzed via Sholl analysis and measured for (A) mean dendritic length (μm), (B) total nodes, (C) cell body area (μm^2^). Data are presented as box and whisker plots in which the box indicates the 25^th^ (lower) to 75^th^ (upper) quartiles; the middle horizontal bar, the median; whiskers, the minimum and maximum values; and dots, the individual values (n = 8-9 animals per group, 5-6 neurons per animal). Data were analyzed for dose effects within sex using mixed effects model. **P* ≤ 0.05 compared to vehicle control.

**Supplemental Figure 2**. ***PCB 11 subtly altered the dendritic arborization of pyramidal neurons in the mouse hippocampus at P21.*** Images of Golgi-stained neurons were obtained from a spinning disc confocal microscope (VT-1000, Leica, Solms, Germany) at 40x magnification. (A) Sholl plots of the dendritic complexity, determined as the number of dendritic intersections at each Sholl ring. The dendritic arbors of male and female neurons were traced using the Neurolucida program (version 11, MBF Bioscience, Williston, VT) and tracings were analyzed using Sholl analysis algorithm in the Neurolucida software. (B) For each Sholl plot, the total area under the curve (AUC) (0-230μm), the proximal AUC (0-70μm), and the distal AUC (71-230μm) were quantified. Tracings were further analyzed to determine (C) the total number of primary dendrites, (D) the total dendrite length for each neuron (μm), (E) the total number of terminal ends per neuron, (F) the mean dendritic length (μm) of the primary dendrite, (G) the total number of nodes (aka branchpoints), and (H) the cell body area (μm^2^). Data are presented as box and whisker plots in which the box indicates the 25^th^ (lower) to 75^th^ (upper) quartiles; the middle horizontal bar, the median; whiskers, the minimum and maximum values; and dots, the individual values (n = 7-8 animals per group, 5-6 neurons per animal). Data were analyzed for dose effects within sex using mixed effects model. **P* ≤ 0.05 compared to vehicle control.

**Supplementary Figure 3.** *PCB 11 did not significantly alter microglial number or phagocytic phenotype at P21.* (A) Representative photomicrographs of Iba1 (green) and CD68 (red) immunoreactivity in the CA3 region of the hippocampus of vehicle and PCB 11-exposed male and female mice (bar = 100 μm). Sections were counterstained with DAPI (blue) to identify cell nuclei. The number of Iba1 (B, D, F, H) and CD68 (C, E, G, I) immunopositive cells per mm^2^ was quantified in the dentate gyrus (B, C), the CA1 of the hippocampus (D, E), the CA3 of the hippocampus (F, G), and the somatosensory cortex (H, I). Data are presented as box and whisker plots in which the box indicates the 25^th^ (lower) to 75^th^ (upper) quartiles; the middle horizontal bar, the median; whiskers, the minimum and maximum values; and dots, the individual values (n = 3-8 animals per group). Data were analyzed using one-way ANOVA within sex with *post hoc* Dunnet’s test, **P* < 0.05 compared to vehicle control.

**Supplemental Figure 4**. *PCB 11 did not dysregulate neurogenesis at P4 in male or female weanlings.* Neurogenesis was measured via immunohistochemical analyses of brain tissue collected at P4. Samples were immunostained with Ki67 (mitotic cells), Doublecortin (DCX) (migrating neurons), and NeuN (mature neurons), and counterstained with DAPI (blue) to identify nuclei. The number of Ki67-positive cells was quantified in the subgranular zone of the dentate gyrus (DG) (A) and the granular zone of the DG (D). (B, E) Migrating neurons were quantified as the percentage of area immunopositive for DCX within each brain region. NeuN immunopositive cells were quantified in the DG (C). Data are presented as box and whisker plots in which the box indicates the 25^th^ (lower) to 75^th^ (upper) quartiles; the middle horizontal bar, the median; whiskers, the minimum and maximum values; and dots, the individual values (n = 6-8 animals per group). Data were analyzed for dose effects using one-way ANOVA within sex with *post hoc* Dunnet’s test.
